# Antioxidant Activity and Anti-Adipogenic Effects of Wild Herbs Mainly Cultivated in Korea

**DOI:** 10.3390/molecules181012937

**Published:** 2013-10-17

**Authors:** Young-Jun Lee, Dan-Bi Kim, Jong Seok Lee, Ju-Hyun Cho, Bong Kyun Kim, Hyeon-Son Choi, Boo-Yong Lee, Ok-Hwan Lee

**Affiliations:** 1 Department of Food Science and Biotechnology, Kangwon National University, Chuncheon 200-701, Korea; E-Mails: hslyj02@gmail.com (Y.-J.L.); danbekim22@nate.com (D.-B.K.); jongseoklee78@gmail.com (J.S.L.); 2 Hurum Central Research Institute, Ochang 363-883, Korea; E-Mails: dusvnd608@hurum.co.kr (J.-H.C.); kp2307@hurum.co.kr (B.K.K.); 3 Department of Food Science and Biotechnology, CHA University, Seongnam 463-836, Korea; E-Mails: choice120@gmail.com (H.-S.C.); bylee@cha.ac.kr (B.-Y.L.)

**Keywords:** wild herb, antioxidant assay, 3T3-L1, total phenolic content, correlation coefficients

## Abstract

Wild herbs, which are edible plants that grow in mountainous areas, have diverse biological effects such as anti-obesity and anti-cancer activities. The aim of this study was to evaluate the total phenolic and flavonoid contents as well as the antioxidant activity of methanol extracts of *Aster scaber*, *Ligularia fischeri*, *Kalopanax pictus*, *Codonopsis lanceolata*, and *Cirsium setidens* and to assess their effects on lipid accumulation and reactive oxygen species (ROS) production during adipogenesis of 3T3-L1 cells. The results revealed that among the five studied wild herb extracts, *Ligularia fischeri* showed the highest total phenolic contents (215.8 ± 14.2 mg GAE/g) and *Aster scaber* showed the highest total flavonoid content (103.9 ± 3.4 mg RE/g). Furthermore, *Aster scaber* and *Ligularia fischeri* extracts showed higher antioxidant activity than the other wild herbs. Regarding anti-adipogenic activity, the *Cirsium setidens* extract significantly inhibited lipid accumulation (~80%) and ROS production (~50%) during adipogenesis of 3T3-L1 cells compared with control cells. These results suggest that wild herbs could be used for the development of functional foods as well as health promoting and pharmaceutical agents.

## 1. Introduction

The presence of an excessive amount of free radicals causes the production of reactive oxygen species (ROS) within the cells [1]. Interestingly, the body has developed various defense mechanisms against free radical-induced oxidative stress, including preventative mechanisms, repair mechanisms, physical defenses and antioxidant defenses [2]. However, recently, the overproduction of ROS by endogenous or external factors such as tobacco smoke, certain pollutants, organic solvents, and pesticides has led to an unbalanced system of antioxidant protection in the body [3]. ROS in a system exceeds the system’s antioxidant capacity to neutralize and eliminate them plays an important role in the disturbance of health and the pathogenesis of various diseases [4].

In the past, synthetic antioxidants, including butylated hydroxytoluene (BHT), butylated hydroxyanisole (BHA) and tertiary butylhydroquinone (TBHQ) were often used in the food industry because they retard undesirable oxidative changes [5,6]. However, in recent years, the use of natural antioxidants derived from natural substances such as plants has attracted considerable interest due to concerns about the safety of synthetic antioxidants [7,8]. Wild herbs are defined as any part of an edible plant that grows in mountainous areas. Recently, wild herbs have also been identified as sources of various phytochemicals, including phenolic compounds, which possess a wide range of biological effects [9,10]. Phenolic and flavonoid compounds are the products of secondary metabolism in plants. They constitute some of the most widespread compounds in the plant kingdom: more than 8,000 are known, and they all have different chemical structures and activities [11]. Significantly, phenolic compounds from plants are known to have potent antioxidant activity. 

Several methods have been developed to evaluate the antioxidant capacity of natural substances, including 2,2-dipheny-l-picrylhydrazyl (DPPH), ferric reducing antioxidant capacity (FRAP), 2,2'-azinobis-3-ethylbenzothiazoline-6-sulphonate (ABTS), oxygen radical absorbance capacity (ORAC) and nitroblue tetrazolium (NBT) assays. Furthermore, recent literature suggests that preadipocyte differentiation has become an intense area of research in recent years and has been studied primarily using *in vitro* models of adipogenesis, including the 3T3-L1 cell line, which is one of the most well-characterized and reliable [12,13,14]. When testing the antioxidant activity of samples, various anti-oxidant assay yields different activities because of their different reaction mechanisms and specific compounds. Therefore, various assays are required to evaluate the *in vitro* antioxidant activity of complex mixtures such as plant extracts [15].

The aim of the present study was to determine the total phenolic content, total flavonoid content, antioxidant effect and anti-adipogenic activity of several wild herbs that are mainly cultivated in Korea (*Aster scaber*, *Ligularia fischeri*, *Kalopanax pictus*, *Codonopsis lanceolata*, and *Cirsium setidens*) and to investigate the correlation coefficients between bioactive compounds and their biological activities.

## 2. Results and Discussion

### 2.1. Total Phenolic and Flavonoid Contents

Plants are rich in variety of phytochemicals such as phenolics and flavonoids, which provide health benefits [16]. Significantly, many studies suggest that these compounds are important antioxidant substances that act as reducing agents, singlet oxygen quenchers or electron donors with chelating properties [17,18]. Thus, we determined the total phenolic and flavonoid contents of methanol extracts of five wild herbs that are mainly cultivated in Korea. As shown in Table 1, among the five wild herb extracts, *Ligularia fischeri* showed the highest total phenolic content (215.8 ± 14.2 mg GAE/g), and *Aster scaber* showed the highest total flavonoid content (103.9 ± 3.4 mg RE/g). Furthermore, the total phenolic contents in wild herb extracts decreased in the following order: *Ligularia fischeri* > *Aster scaber* > *Kalopanax pictus* > *Cirsium setidens* > *Codonopsis lanceolata*. The flavonoid contents in wild herb extracts decreased in the following order: *Aster scaber* > *Ligularia fischeri* > *Cirsium setidens* > *Kalopanax pictus* > *Codonopsis lanceolata*.

**Table 1:** Total phenolic and flavonoid contents of methanol extracts of five wild herbs.

Extract	Total phenolic contents (mg GAE/g)	Total flavonoid contents (mg RE/g)
*Aster scaber* extract	183.5 ± 4.0 ^b^	103.9 ± 3.4 ^a^
*Ligularia fischeri* extract	215.8 ± 14.2 ^a^	86.9 ± 3.8 ^b^
*Kalopanax pictus* extract	75.4 ± 8.3 ^c^	30.5 ± 4.7 ^c^
*Codonopsis lanceolata* extract	14.9 ± 6.1 ^e^	3.8 ± 3.7 ^e^
*Cirsium setidens* extract	55.4 ± 7.3 ^d^	58.3 ± 5.3 ^d^

All values are presented as the means ± SD. Values with different letters indicate statistically significant differences among groups at *p* < 0.05 by one-way ANOVA.

### 2.2. Antioxidant Activity

The antioxidant activity of the wild herbs was analyzed using *in vitro* methods such as DPPH radical scavenging, ferric reducing antioxidant power (FRAP) activity, ABTS radical cation scavenging activity, reducing power and oxygen radical absorbance capacity (ORAC).

**Figure 1:**
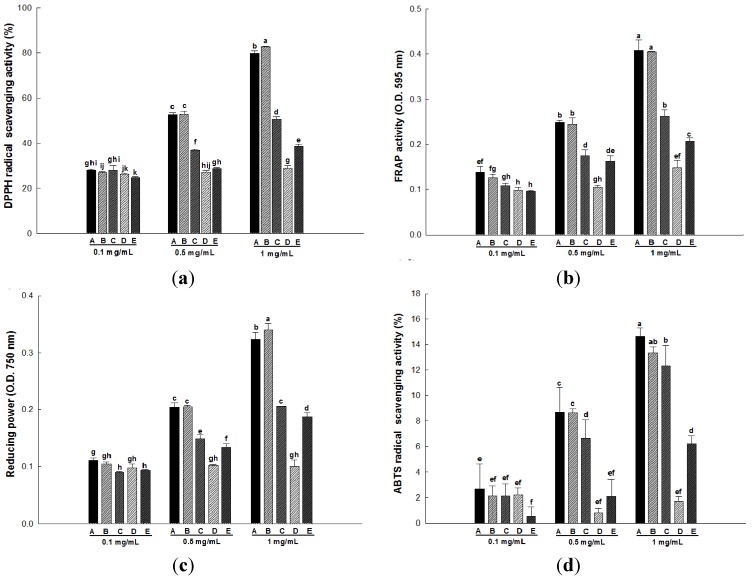
DPPH radical scavenging activity (**a**), FRAP activity (**b**), reducing power (**c**) and ABTS radical scavenging activity (**d**) of five wild herb extracts at various concentrations. A, *Aster scaber*; B, *Ligularia fischeri*; C, *Kalopanax pictus*; D, *Codonopsis lanceolata*; E, *Cirsium setidens*. All values are presented as the means ± SD. Bars with different letters indicate statistically significant differences among groups at *p* < 0.05 by one-way ANOVA.

The DPPH method is one of the most extensively used antioxidant assays because it is a quick, reliable and reproducible method that can be used for examining the general antioxidant activity of natural substances as well as plant extracts *in vitro* [19,20]. This method is based on the ability of DPPH to accept an electron or hydrogen radical from an antioxidant to form a stable diamagnetic molecule, triggering a color change at different concentrations (from purple to yellow) that can be measured at 517 nm [21]. Our results show that the DPPH radical scavenging activity of wild herb extracts was increased in a dose-dependent manner. Importantly, the activity of the *Ligularia fischeri* extract was significantly higher (*p* < 0.05) than the activities of the other wild herb extracts (Figure 1a).

The FRAP assay, which provides fast reproducible results, measures the ability of an antioxidant to reduce the ferric tripyridyltriazine (Fe^3+^-TPTZ) complex and produce the ferrous tripyridyltriazine (Fe^2+^-TPTZ) complex, which is blue in color and can be detected at 593 nm [22]. In this study, the FRAP activity of wild herb extracts was increased in a dose-dependent manner. No significant difference was observed between the FRAP activity of *Aster scaber* and *Ligularia fischeri* extracts, and these activities were markedly higher than those of other wild herb extracts at concentrations of 0.5 and 1 mg/mL (Figure 1b).

Reducing power is usually associated with the presence of reductones, which have been shown to exert antioxidant action by breaking the free radical chain via the donation of a hydrogen atom [23]. Furthermore, reductones also reportedly react with certain precursors of peroxide, thus preventing peroxide formation. Reducing power has been investigated based on the reduction of the ferricyanide complex (Fe^3+^) to the ferrous (Fe^2+^) form by reducers such as antioxidants [24]. As shown in Figure 1c, the reducing power of the five wild herb extracts (at a concentration of 1 mg/mL) decreased in the following order: *Aster scaber* > *Ligularia fischeri* > *Kalopanax pictus* > *Cirsium setidens* > *Codonopsis lanceolata*.

The mechanisms of antioxidant action in the ABTS assay differed from those in the FRAP and reducing power assays. The FRAP assay employs an electron donor, and the oxidation chain reaction is terminated by reducing the oxidized intermediates to their stable forms; however, the ABTS assay employs a hydrogen donor, and the oxidation process is terminated by the conversion of free radicals into more stable products [25]. Figure 1d shows that the ABTS radical scavenging activity of the methanol extracts of five wild herbs was increased in a dose-dependent manner. Furthermore, as mentioned above, *Aster scaber* and *Ligularia fischeri* extracts performed better than the other wild herb extracts with respect to their ABTS radical scavenging activity. Particularly, the ABTS radical scavenging activity of the *Aster scaber* extract was increased by ~8-fold compared with that of the *Codonopsis lanceolata* extract.

**Figure 2:**
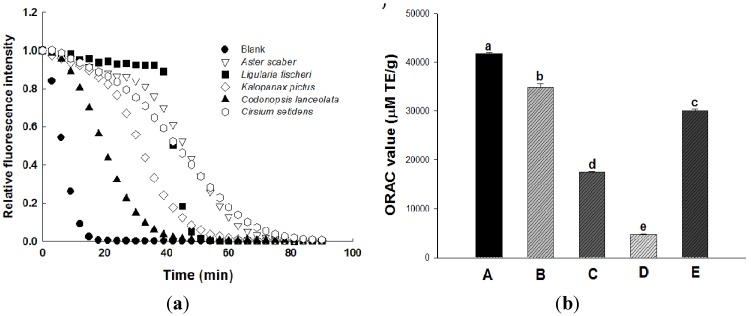
Radical scavenging activity of wild herb extracts as measured by ORAC. (**a**) Decay curve of fluorescence in the presence or absence of samples. (**b**) Radical scavenging activity of wild herb extracts expressed as Trolox equivalents. A, *Aster scaber*; B, *Ligularia fischeri*; C, *Kalopanax pictus*; D, *Codonopsis lanceolata*; E, *Cirsium setidens*. All values are presented as the means ± SD. Bars with different letters indicate statistically significant differences among groups at *p* < 0.05 by one-way ANOVA.

The ORAC assay is based on the peroxyl radical scavenging capacity induced by AAPH at 37 °C using fluorescein as a fluorescence probe [26]. In the ORAC assay, the effectiveness of an antioxidant is measured based on changes in the areas under the curve (AUC) of the fluorescence decay kinetics profiles of each sample. Figure 2a shows the kinetic profiles of fluorescein consumption in the absence or presence of five wild herb extracts. As shown in Figure 2, fluorescein was efficiently protected by the samples. Among the five wild herbs, the highest ORAC value was observed for the *Aster scaber* extract (41,638 ± 282 µM TE/g) and the lowest was observed for the *Codonopsis lanceolata* extract (4,749 ± 93 µM TE/g). The order of ORAC values was as follows: *Aster scaber* > *Ligularia fischeri* > *Cirsium setidens* > *Kalopanax pictus* > *Codonopsis lanceolata*.

### 2.3. Effect of Wild Herb Extracts on the Cell Viability of 3T3-L1 Preadipocytes

**Figure 3:**
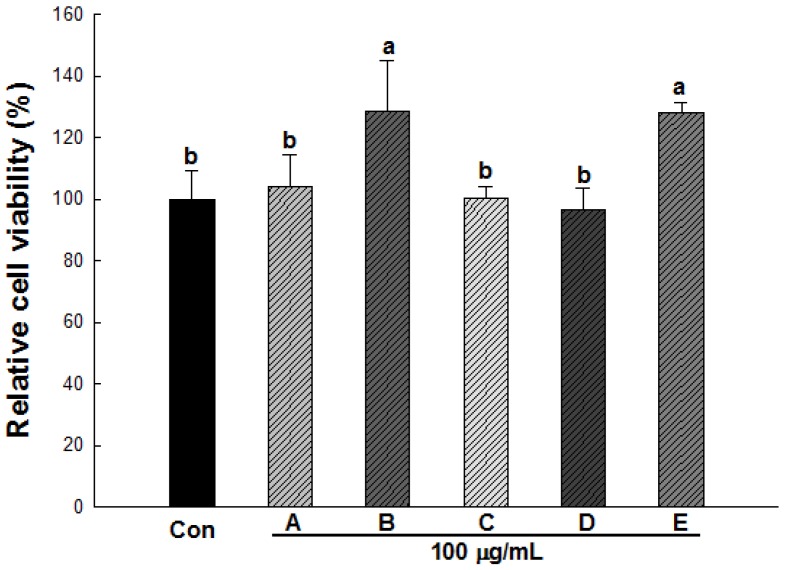
Effect of wild herb extracts on the cell viability of 3T3-L1 preadipocytes. Cell viability was determined using the XTT assay. Con, control cells; A, *Aster scaber*; B, *Ligularia fischeri*; C, *Kalopanax pictus*; D, *Codonopsis lanceolata*; E, *Cirsium setidens*. All values are presented as the means ± SD. Bars with different letters indicate statistically significant differences among groups at *p* < 0.05 by one-way ANOVA.

To evaluate the cytotoxicity of methanol extracts of five wild herbs, 3T3-L1 adipocytes were treated with these extracts at a concentration of 100 μg/mL for 24 h. As shown in Figure 3, the extracts did not cause any cytotoxicity at a 100 μg/mL concentration, and no changes in cell morphology were observed by microscopic analysis (data not shown). We note that the increased cell viability that was observed upon treatment with *Ligularia fischeri* and *Cirsium setidens* extracts may have been due to the color that was present in the media of each extract, which may have been detected by spectrophotometrical measurements. Here, we show that these extracts did not cause cytotoxicity throughout their entire concentration range up to 100 μg/mL; thus a concentration of 100 μg/mL was used in the experiments described below.

### 2.4. Lipid Accumulation and ROS Production in 3T3-L1 Cells

The effects of the methanol extracts of five wild herbs on lipid accumulation and ROS production were determined by Oil Red O staining and the NBT assay during the differentiation of 3T3-L1 cells. Two-day post-confluent 3T3-L1 preadipocytes (day 0) were treated with methanol extracts of five wild herbs at 100 μg/mL every 2 days for 8 days. When the preadipocytes were differentiated into adipocytes via the application of MDI cocktail, morphological changes were observed due to the accumulation of lipid droplets in the cytoplasm. 

**Figure 4:**
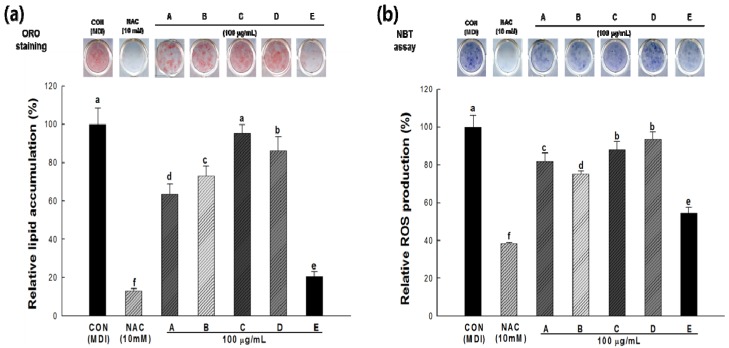
Effect of the five wild herb extracts on lipid accumulation (**a**) and reactive oxygen species (ROS) production (**b**) in 3T3-L1 cells during the adipogenesis. Accumulated lipids were stained with Oil Red O reagent and measured based on the absorbance at 490 nm. ROS production was assessed based on the formation of dark-blue formazan and determined by the NBT assay at a wavelength of 570 nm. CON, control cells which were differentiated with MDI; NAC, positive control cells which were differentiated with MDI in the presence of NAC; A–E, cells which were differentiated with MDI in the presence of each sample; A, *Aster scaber*; B, *Ligularia fischeri*; C, *Kalopanax pictus*; D, *Codonopsis lanceolata*; E, *Cirsium setidens*. All values are presented as the means ± SD. Bars with different letters indicate statistically significant differences among groups at *p* < 0.05 by one-way ANOVA.

Figure 4a shows that the lipid accumulation was strongly induced in control differentiated 3T3-L1 adipocytes, whereas the cells treated with wild herb extracts showed significantly decreased intracellular lipid accumulation compared with the control cells, except for cells treated with *Kalopanax pictus* extracts. Among the five wild herbs, the *Cirsium setidens* extract had the greatest anti-adipogenic effects; 3T3-L1 cells treated with this extract showed significantly reduced lipid accumulation (~80%) compared with control cells. In this study, N-acetylcysteine (NAC), a well-known positive control during adipogenesis, caused a significant reduction in lipid accumulation (~85%) in 3T3-L1 cells [27]. Many studies have reported that obesity may induce oxidative stress in humans and mice [13]. It is important to note that the lipid accumulation decreased in 3T3-L1 cells because lipid accumulation during adipogenesis is positively related to ROS production.

The NBT assay is a well-established technique that is used to quantify cellular oxidative metabolism [28]. The production of dark-blue formazan, which represents intracellular ROS production, was significantly decreased in the cells treated with wild herb extracts when compared with control cells (Figure 4b). As mentioned above, the *Cirsium setidens* extract had the highest antioxidant activity (~50%) among the five wild herb extracts. Furthermore, a strong correlation was observed between lipid accumulation and ROS production in 3T3-L1 cells (*R* = 0.935, *p* < 0.05). These results suggest that lipid accumulation increases in parallel with ROS production in adipocytes.

### 2.5. Correlation Coefficients (R) between Bioactive Compounds and Their Biological Activity

Table 2 shows the correlation coefficients among total phenols, total flavonoids and antioxidant capacity as well as lipid accumulation and ROS production. Strong correlations were observed between the total phenolic contents (TPC) and antioxidant capacity including DPPH (*R* = 0.991, *p* < 0.01), FRAP (*R* = 0.984, *p* < 0.01) and reducing power (*R* = 0.985, *p* < 0.01) assays. On the other hand, TPC was only moderately correlated with both ABTS (*R* = 0.847, *p* > 0.05) and ORAC (*R* = 0.825, *p* > 0.05) assays and poorly correlated with the NBT (*R* = 0.108, *p* > 0.05) assay, which may be caused by the different reaction mechanisms of the antioxidant activity determination methods [29].

**Table 2:** Correlation coefficients (R) among total phenolics, total flavonoids and antioxidant capacity as well as lipid accumulation and ROS production in 3T3-L1 cells.

	TPC ^a^	TFC ^b^	DPPH	FRAP	Reducing power	ABTS	ORAC	Lipid accumulation	ROS production
**TPC**	1	0.890 ^*^^c^	0.991 ^**^	0.984 ^**^	0.985 ^**^	0.847	0.825	0.017	0.108
**TFC**		1	0.882 ^*^	0.899 ^*^	0.930 ^*^	0.774	0.987 ^**^	0.379	0.419
**DPPH**			1	0.998 ^**^	0.985 ^**^	0.898 ^*^	0.816	0.082	0.035
**FRAP **				1	0.988 ^**^	0.914 ^*^	0.840	0.053	0.056
**Reducing power**					1	0.901 ^*^	0.890 ^*^	0.070	0.0203
**ABTS**						1	0.753	0.142	0.021
**ORAC**							1	0.485	0.536
**Lipid accumulation**								1	0.935 ^*^
**ROS production**									1

^(^^a^^)^ Total phenolic contents; ^(^^b^^)^ Total flavonoid contents; ^(^^c^^)^ * The correlation coefficients is significant at the 0.05 level; ** the correlation is significant at the 0.01 level.

Total flavonoid contents (TFC) correlated with DPPH (*R* = 0.882, *p* < 0.05), FRAP (*R* = 0.899, *p* < 0.05) and reducing power (*R* = 0.930, *p* < 0.05) assays. These correlations were lower than the correlation between TPC and antioxidant capacity (Table 2). Chun [30] and Kim [31] reported that antioxidant capacity as measured by DPPH and ABTS were more strongly correlated with TPC versus TFC. The antioxidant capacity results determined by DPPH, FRAP and reducing power assays were similar to these previous results; however, the correlation between TFC and ORAC was higher than that of TPC and ORAC (Table 2). Furthermore, some antioxidant capacity assays (DPPH, FRAP, reducing power and ABTS) were significantly correlated with each other, whereas ORAC and NBT were weakly correlated with other antioxidant capacity assays (Table 2). This result may have occurred because the ORAC assay takes the kinetic action of antioxidants into account. Whereas other methods such as DPPH, FRAP, reducing power and ABTS are colorimetric assays. Roy reported that, in general, the antioxidant capacity of a flavonoid can be attributed to the number of -OH groups; therefore, radical scavenging activity as measured by the DPPH assay has a strong correlation with the number of -OH groups [32]. However, a weak correlation was observed between the number of -OH groups and the corresponding ORAC value of catechins. In addition, similar to EGCG, the flavonoids have an -OH group in the C3 position; EGCG has potent DPPH radical scavenging activity but decreased ORAC activity. Cao suggested that the number of -OH groups in the A ring and B ring of flavonoids was strongly correlated with their ORAC values [18]. Therefore, due to different reaction mechanisms, the evaluation of antioxidant capacity is a rather difficult task when a single method is selected. For that reason, several methods should be used to elucidate the full antioxidant activity profile of samples.

## 3. Experimental

### 3.1. Materials

Methanol extracts of five wild herbs that are mainly cultivated in Korea, including *Aster scaber*, *Ligularia fischeri*, *Kalopanax pictus*, *Codonopsis lanceolata*, and *Cirsium setidens*, were purchased from the Korea Research Institute of Bioscience and Biotechnology (KRIBB, Taejeon, Korea). These extracts were prepared as follows: dried samples (100 g) were extracted with 1,000 mL of methanol at 45 °C using an ultrasonic cleaner (Branson Ultrasonics Corporation, Danbury, CT, USA) for 96 h. The extracted materials were filtered and concentrated with a rotary evaporator (N-1000SWD, Eyela, Tokyo, Japan) at 45 °C for 24 h. The extracted materials were freeze-dried using a freeze dryer (Biotron, BPuchon, Korea). The extracts were dissolved in DMSO or ethanol used in the experiment. Oil Red O (ORO), 3-isobutyl-1-methylxanthine (IBMX), dexamethasone (DEX), isopropanol, rutin, ascorbic acid, gallic acid, 1,1-diphenyl-2-picryl hydrazyl radical (DPPH), N-acetyl-L-cysteine (NAC), trichloroacetic acid (TCA), 2,2'-azino-bis(3-ethylbenzothiazoline-6-sulfonic acid) diammonium salt (ABTS), Folin-Ciocalteu phenol reagent, potassium ferricyanide, sodium carbonate, 2,4,6-tris(2-pyridyl)-s-triazine (TPTZ), potassium persulfate, 6-hydroxy-2,5,7,8-tetramethylchroman-2-carboxylic acid (Trolox), insulin, acetic acid, 2,2-azobis(2-amidino propane) dihydrochloride (AAPH), fluorescein sodium salt, and sodium nitrite (NaNO2) were procured from Sigma (St. Louis, MO, USA). Dulbecco’s modified Eagle’s medium (DMEM), bovine serum (BS), fetal bovine serum (FBS), penicillin-streptomycin (P/S), phosphate-buffered saline (PBS) and trypsin-EDTA were purchased from Gibco (Gaithersburg, MD, USA).

### 3.2. Total Phenolic and Flavonoid Contents

The total phenolic contents of methanol extracts of wild herbs were determined by the method of Gutfinger with some modifications [33]. The sample solution (1 mL) was placed in a test tube with 10% (v/v) Folin-Ciocalteu reagent (1 mL), and a 2% sodium carbonate solution (1 mL) was added after the mixture was incubated at 25 °C for 1 h. The absorbance was measured at 750 nm, and the calibration curve was prepared using gallic acid. The results are expressed in mg of gallic acid equivalent (mg GAE)/g.

The total flavonoid contents of methanol extracts of wild herbs were determined according to the method of Moreno [34]. The sample solution (0.5 mL) was mixed with 1.5 mL ethanol (95%, v/v), 0.1 mL of aluminum chloride (10%, w/v), 0.1 mL of potassium acetate (1 M) and distilled water. After incubation at room temperature for 30 min, the absorbance was determined at 415 nm, and a calibration curve was prepared using rutin. The results are expressed in mg of rutin equivalents (mg RE)/g.

### 3.3. DPPH Radical Scavenging Assay

The DPPH assay was performed according to the method of Chu with some modifications [35]. A DPPH solution (0.4 mM) in anhydrous ethanol was stirred for 30 min, and the absorbance of the solution was adjusted to 1.0 ± 0.1 at 490 nm. Then, 0.2 mL of the sample (or a control) was mixed with 0.8 mL of DPPH solution and incubated for 10 min in the dark at room temperature. The decrease in absorbance was measured at 490 nm, and the radical scavenging activity was calculated and expressed as a percentage using the following formula:

DPPH radical scavenging activity (%) = [1 − (Atest/Bcontrol)] × 100 (1)

### 3.4. FRAP Assay

The ferric reducing antioxidant power (FRAP) assay was carried out according to the method of Benzie with some modifications [22]. The FRAP reagent was prepared by mixing acetate buffer (300 mM, pH 3.6), 10 mM TPTZ solution (in 40 mM HCl) and 20 mM FeCl3∙6H2O at a ratio of 10:1:1 (v/v), respectively. The FRAP reagent was prepared fresh and warmed to 37 °C in a water bath prior to use. The sample (50 μL) was mixed with 150 μL of distilled water and 1.5 mL of the reagent, and the mixture was incubated at 37 °C for 4 min. The absorbance of the reaction mixture was measured at 593 nm.

### 3.5. Reducing Power

The reducing power assay was performed according to the method of Oyaizu [24]. Various concentrations of sample solutions were mixed with 2.5 mL of sodium phosphate buffer (pH 6.6) and 2.5 mL of potassium ferricyanide (1%, v/v). The mixtures were incubated at 50 °C for 20 min. After the addition of 2.5 mL of trichloroacetic acid (10%, w/v), the mixture was centrifuged at 1,790 × g for 10 min. A 2.5-mL aliquot of the supernatant was mixed with 2.5 mL of distilled water and 0.5 mL of ferric chloride (0.1%, w/v), and the absorbance was measured at 750 nm.

### 3.6. ABTS Radical Scavenging Activity

The ABTS assay was based on the method of Re with some modifications [36]. ABTS (7 mM, w/v) dissolved in water was mixed with 2.45 mM potassium persulfate, and the ABTS-potassium persulfate solution (1:0.5, v/v) was incubated in the dark at 20 °C for 16 h. The ABTS^+^ solution was diluted with ethanol until an absorbance of 0.75 ± 0.02 was achieved at 750 nm. Then, 1 mL of ABTS^+^ solution was added to 10 μL of the sample solutions at different concentrations. The absorbance of the mixture was measured at 734 nm after 6 min. ABTS radical scavenging activities were calculated and expressed as a percentage using the following formula:

ABTS radical scavenging activity (%) = [1 − (Atest/Acontrol)] × 100 (2)

### 3.7. ORAC Assay

The ORAC assay was based on the method of Ou with some alterations [26]. The experiment was conducted in 75 mM phosphate buffer (pH 7.4) at 37 °C. A 25-μL sample and 150 μL of 40 nM fluorescein (FL) were dispensed into 96-well plates. Then, 25-μL aliquots of AAPH (18 mM), which was preincubated at 37 °C for 15 min, were subsequently dispensed into each well, and the plate was immediately placed in the fluorescence microplate reader (Spectramax GEMINI EM, Molecular Devices, Sunnyvale, CA, USA). The reader was programmed to record the fluorescence of FL every 3 min for 90 min at emission and excitation wavelengths of 535 nm and 485 nm, respectively. Trolox, a water-soluble analog of vitamin E, was used as a standard, and a blank was measured that contained phosphate buffer in place of the sample. The ORAC values were calculated using a Trolox calibration curve and the area under the fluorescence decay curve. ORAC values were expressed as Trolox equivalents in μmol/mL.

Area under the curve (AUC) = 1 + f1/f0 + f2/f0 + f3/f0 +f4/f0 + ...f/31/f0(3)

### 3.8. Cell Culture

3T3-L1 preadipocytes obtained from the American Type Culture Collection (CL-173, ATCC, Manassas, VA, USA) were cultured, maintained, and differentiated as described by Lee [37]. In brief, the cells were plated and grown in DMEM with 3.7 g/L sodium bicarbonate, 1% P/S and 10% BCS. Adipocyte differentiation was induced when post-confluent cells were treated with 10% FBS and a hormonal cocktail (MDI) consisting of 0.5 mM IBMX, 1.0 μM DEX, and 1.67 μM insulin for 2 days. Two days after the initiation of differentiation, the culture medium was replaced with DMEM supplemented only with 1.67 μM insulin and 10% FBS. This medium was then replenished every 2 days. For the treatments, 2-day post-confluent cells were differentiated with MDI in the presence of samples (100 µg/mL) or NAC (10 mM ≈ 1.63 mg/mL).

### 3.9. XTT Assay

Cell proliferation was detected by the 2,3-bis (2-methoxy-4-nitro-5-sulfophenyl)-2H-tetrazolium-5-carboxanilide inner salt (XTT) assay (WelGene, Seoul, Korea). When the cells were cultured to the log phase, they were seeded on a 96-well plate (1 × 10^5^ cells/well) for 24 h. The cells were divided into a control group and treatment groups at the concentrations indicated. Absorbance (A) was determined with an enzyme calibrator at 450 nm. 

Cell viability = (A of study group/A of control group) × 100%(4)

### 3.10. NBT Assay

3T3-L1 preadipocytes were grown to confluence and induced to differentiate into adipocytes as previously described [37]. ROS production was detected using the NBT assay. NBT can be reduced by ROS to formazan, which is dark blue and insoluble [13]. On day 8 after induction, the cells were incubated for 90 min in PBS containing 0.2% NBT. Formazan was dissolved in 50% acetic acid, and the absorbance was determined at 570 nm.

### 3.11. Oil Red O Staining

The extent of differentiation reflected by the amount of lipid accumulation was determined on day 8 using Oil red O staining. In brief, the cells were fixed in 10% formaldehyde in distilled water for 1 h, washed with distilled water, and dried completely. The cells were stained with 0.5% Oil red O dissolved in 60% isopropanol for 30 min at room temperature, washed four times in water, and dried. Differentiation was also monitored under a microscope and quantified via elution with isopropanol and obtaining optical density (OD) measurements at 490 nm [37].

### 3.12. Statistical Analysis

All measurements were repeated three times. The results are expressed as the mean values ± standard deviation. The results were statistically analyzed by ANOVA and Duncan’s multiple range tests. Statistical significance was set at *p* < 0.05 (SAS-Institute, Cary, NC, USA, 1998).

## 4. Conclusions

In this study, the total phenolic contents, flavonoid contents and antioxidant activities of methanol extracts of five wild herbs cultivated in Korea were investigated, and their effects on lipid accumulation and ROS production during adipogenesis of 3T3-L1 cells was examined. We used various *in vitro* antioxidant activity measurement methods to determine that these wild herb extracts have significant antioxidant activity. Significantly, *Aster scaber* and *Ligularia fischeri* extracts showed greater antioxidant activity compared with the other wild herbs. Furthermore, among the studied wild herbs, the *Cirsium setidens* extract had the highest anti-adipogenic effects. Based on the results described above, we conclude that wild herbs are an excellent natural source of antioxidants and can be utilized to develop functional foods as well as health promoting and pharmaceutical agents.
